# Case report: accelerated cathodal HD-tDCS over the right dorsolateral prefrontal cortex in hoarding disorder

**DOI:** 10.3389/fnhum.2023.1327811

**Published:** 2024-03-11

**Authors:** Jerome Brunelin, Cécilia Neige, Julien Eche, Filipe Galvao, Rémy Bation, Marine Mondino

**Affiliations:** ^1^Centre Hospitalier Le Vinatier, Bron, France; ^2^Université Claude Bernard Lyon 1, CNRS, INSERM, Centre de Recherche en Neurosciences de Lyon CRNL U1028 UMR5292, PSYR2, Bron, France; ^3^Hospices Civils de Lyon, Service de Psychiatrie de Liaison, Groupement Hospitalier Sud, Pierre Benite, France

**Keywords:** case report, high-definition transcranial direct current stimulation, hoarding disorder, depression, anxiety, DLPFC

## Abstract

Hoarding disorder is an under-recognized condition characterized by the excessive acquisition of possessions and difficulty in disposing of them, which can have dramatic consequences. As hoarding disorder is difficult to treat and associated with high levels of disability in all areas of functioning, there appears to be a critical need to develop novel, tailored therapeutic strategies. Non-invasive brain stimulation techniques hold promise as potential therapeutic interventions for various psychiatric conditions and as a tool to modulate impulsivity when applied over the dorsolateral prefrontal cortex (DLPFC). Therefore, we hypothesized that delivering accelerated cathodal high-definition direct transcranial stimulation (HD-tDCS) over the right DLPFC could be a suitable approach to alleviate symptoms in patients with hoarding disorder. In a case report, we observed beneficial clinical effects on acquisition and depressive symptoms after 15 sessions of three daily 20-min sessions. Accelerated cathodal HD-tDCS over the right DLPFC appears to be a safe and appropriate intervention for patients with hoarding disorder. However, randomized, sham-controlled trials are needed to further validate these encouraging findings.

## 1 Introduction

Hoarding disorder is a mental health condition characterized by the excessive acquisition of possessions and difficulty in disposing of them, resulting in severely cluttered living spaces that can significantly impact the quality of life. The impact of hoarding goes beyond physical clutter; its symptoms are associated with high levels of disability in all areas of functioning, leading to social exclusion and the inability to live alone in a decent home (Nutley et al., 2022). The accumulation of possessions can lead to the risk of house collapse and fire, with dramatic consequences for the individual and the neighborhood.

Hoarding disorder is a prevalent condition, estimated to affect 2–5% of the population, and has often been associated with obsessive-compulsive disorder (OCD). However, fewer than 20% of people with hoarding disorder actually meet the criteria for an OCD diagnosis (Frost et al., 2011). In addition, rates of comorbid conditions, such as major depressive disorder (MDD) and acquisition-related impulse control disorder are higher in patients with hoarding disorder than in those with OCD.

The overall goals of therapeutic interventions for hoarding disorder focus on reducing clutter, improving decision-making skills, preventing safety hazards, addressing underlying emotional issues, and ultimately improving quality of life. Interventions typically involves a combination of therapeutic approaches, including cognitive-behavioral therapy (CBT) and medication. However, response rates to a standardized course of selective serotonin reuptake inhibitors (SSRIs) combined with CBT are often unsatisfactory (Saxena et al., 2002). The limited response to treatment, coupled with patients’ lack of insight and resistance to change, makes hoarding disorder particularly challenging to treat (Frost et al., 2010). This challenge contributes to the overall burden of the disorder and highlights the need to develop new interventions.

Recently, non-invasive brain stimulation techniques such as transcranial direct current stimulation (tDCS) have emerged as alternative therapeutic interventions for patients with treatment-resistant psychiatric symptoms and as tools to investigate the cognitive functioning, including impulsivity. This approach involves applying tDCS to brain regions that have been shown to play a role in the specific symptoms or cognitive function being addressed. These regions include the motor/premotor areas, the cerebellum, the orbitofrontal cortex and the dorsolateral prefrontal cortex (DLPFC) when targeting symptoms in patients with OCD (Brunelin et al., 2018) and the DLPFC when modulating impulsivity (Brevet-Aeby et al., 2016). Given the overlap between OCD and hoarding disorder, Handrack et al. have recently used a similar approach for a patient with hoarding disorder and kleptomania (Handrack et al., 2018). In a case report, they observed a 24% reduction in hoarding disorder symptoms, as assessed using the Saving Inventory-Revised (SI-R) scale (Frost et al., 2004), following 16 sessions of tDCS applied with the anode to the left DLPFC and the cathode to the right DLPFC.

Recent advances in tDCS technology have paved the way for more precise and focused targeting of brain regions. This refined technique, known as high-definition tDCS (HD-tDCS), employs a setup of multiple smaller electrodes instead of the two larger pad-electrodes of conventional tDCS (Datta et al., 2009). HD-tDCS has shown beneficial effects in patients with OCD and comorbid bipolar disorder (Parlikar et al., 2019) or schizophrenia (Nayok et al., 2021). Based on neuroimaging studies showing increased activity in the right DLPFC in hoarding disorder (Hough et al., 2016), the key role of the right DLPFC in impulsivity (Brevet-Aeby et al., 2016), and the previous case report (Handrack et al., 2018), we proposed that cathodal HD-tDCS over the right DLPFC would be effective in reducing symptoms of hoarding disorder and modulating cortical excitability.

## 2 Material and methods

In November 2022, a 63-year-old female patient with severe and debilitating treatment-resistant hoarding disorder was referred to our non-invasive brain stimulation unit. Over the last few years, she has undergone multiple pharmacological treatments, including SSRIs (fluoxetine 60 mg, paroxetine 60 mg, escitalopram 20 mg), serotonin and noradrenaline reuptake inhibitors (SNRIs) (venlafaxine 150 mg, duloxetine 60 mg), clomipramine 150 mg and augmentation with antipsychotic (aripiprazole 10 mg, risperidone 4 mg) in combination with CBT, with limited to no clinical response. She had no history of suicidality, substance abuse, or relevant somatic conditions. Given the chronic nature of the disease, the resistance of the symptoms, and the significant social consequences of the disorder, we decided to propose a treatment with HD-tDCS. The patient provided written informed consent after receiving a description of the study. Upon admission, she was no longer on medication but was engaged in CBT. She did not meet the Diagnostic and Statistical Manual of Mental Disorders, Fifth Edition (DSM-5) criteria for OCD or MDD and did not exhibit psychotic features. Her brain MRI was normal.

Hoarding symptoms were assessed before HD-tDCS at baseline (D0), after HD-tDCS (D15), at 1-month follow-up (M1) and at 3-month follow-up (M3) using the SI-R (Frost et al., 2004). Mood was assessed using the 10-item Montgomery-Åsberg Depression Rating Scale (MADRS) and the 14-item Hospital Anxiety and Depression Scale (HAD).

Altered cortical excitability is a ubiquitous finding in several psychiatric disorders (Radhu et al., 2013) and can be restored after DLPFC stimulation (Cheng et al., 2022). Therefore, neurophysiological measures were collected at baseline (D0) and after HD-tDCS (D15) using transcranial magnetic stimulation (TMS) (MagVenture, MagPro X100, Denmark) with a 65 mm figure-of-eight coil. Electromyographic recordings of the left first dorsal interosseous (FDI) muscle were made with disposable Ag/AgCl surface electrodes. The optimal stimulation site on the scalp (hotspot) was defined as the site that elicited the largest MEP amplitude in the FDI muscle for a given intensity. This site was marked on a tight-fitting cap that was worn by the patient. Resting motor threshold, corticospinal excitability and cortical Silent Period (cSP) were assessed using single-pulse TMS applied at rest or during a low-force isometric finger contraction. Measures of intracortical inhibition [short-interval intracortical inhibition (SICI), long-interval intracortical inhibition (LICI), and intracortical facilitation (ICF)] were assessed using paired-pulse TMS applied over the right primary motor cortex (see Supplementary Table 1 for stimulation parameters). Twenty Motor Evoked Potentials (MEP) were collected for each condition. Paired-pulses mean MEP amplitude were expressed as a percentage of single-pulses mean MEP amplitude (Neige et al., 2020).

High-definition tDCS (HD-tDCS) sessions were delivered using a Soterix MXN-33 stimulator with a 4 × 1 HD-tDCS ring electrode montage (Soterix Medical, New Jersey, USA). The cathode was placed over the right DLPFC at F4 (−2 mA), surrounded by 4 anodes at AF4, F2, FC4, and F6 (+0.5 mA) according to the international 10/10 EEG system. A ramp-up/down period of 30 s was defined. Three 20-min sessions, spaced 20 min apart, were administered daily over the course of 5 consecutive days, resulting in a total of 15 sessions.

## 3 Results

**FIGURE 1:**
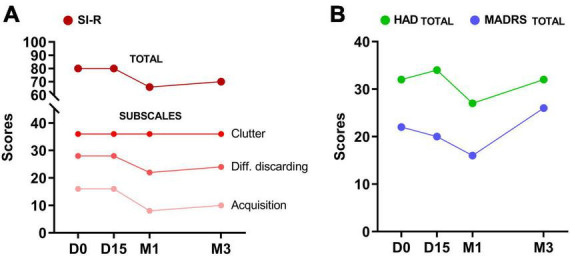
Changes in hoarding symptoms (SI-R total and subscale scores) in **(A)** depressive symptoms (MADRS) and **(B)** mood (HAD) after 15 sessions of HD-tDCS over the right DLPFC (D15), at 1-month (M1) and 3-month (M3) follow-up.

The treatment was well tolerated, and no adverse events were observed. The SI-R total score decreased from 80 at baseline to 66 at M1 (−18.5%). This reduction was mainly in the SI-R excessive acquisition subscale (−50%), while only a modest 22% decrease was observed in the difficulty discarding subscale. No effect was observed on the clutter subscale (Figure 1A). Depressive symptoms decreased by 27% at M1, as indicated by a decrease in MADRS scores from 22 to 16 (Figure 1B). This reduction in depressive symptoms was also observed on the HAD depression subscale with a decrease of 32%, whereas no effect was observed on the anxiety subscale of the HAD. No effects were observed at D15 and mood symptoms appeared to have returned to baseline at M3 (MADRS, HAD). However, a 38% decrease in the SI-R acquisition subscale was still observed at M3 (−12.5% for the SI-R total score).

**FIGURE 2:**
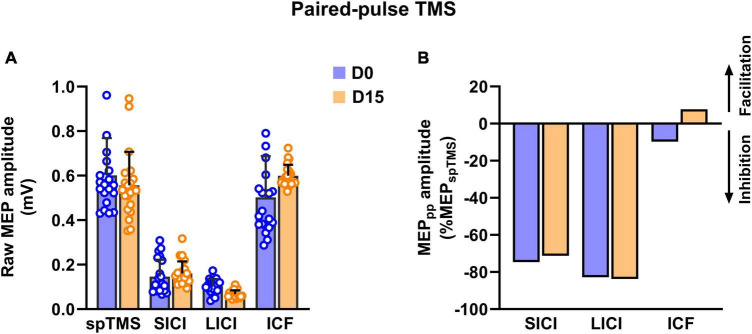
Raw MEP amplitude **(A)** and normalized MEP **(B)** reflecting changes in inhibitory [short-interval intracortical inhibition (SICI)], long-interval intracortical inhibition (LICI) and facilitatory processes [intracortical facilitation (ICF)] processes after 15 sessions of HD-tDCS (D15) over the right DLPFC in a patient with hoarding disorder.

Regarding neurophysiological measures, we observed no changes after HD-tDCS in the resting motor threshold (52% of maximal stimulator output at both D0 and D15), corticospinal excitability (0.58 ± 0.13 mV at D0 *vs.* 0.55 ± 0.15 mV at D15) and no change in cSP duration (96.6 ± 6 ms at D0 *vs.* 95.6 ± 15 ms at D15). Using paired-pulse TMS, we observed no changes in SICI and LICI, whereas a significant increase in intracortical facilitation process was observed (from a clear absence of facilitation at D0: −9.77% to + 7.78% at D15; Figure 2).

## 4 Discussion

The current results highlight the potential of 15 sessions of cathodal HD-tDCS applied using a 4 × 1 electrode montage over the right DLPFC to induce noteworthy improvements in depressive symptoms (mean reduction of 30%) and excessive acquisition (−50%) in a patient with hoarding disorder. However, no substantial effects were observed on anxiety, clutter and difficulty discarding symptoms. The 18.5% reduction in hoarding symptoms was comparable to that observed in the previous case study in hoarding disorder with conventional bifrontal tDCS (−24% on the SI-R) (Handrack et al., 2018). Interestingly, the clinical effects were not observed immediately after the end of the HD-tDCS sessions, but rather manifested at the 1-month follow-up, suggesting a delayed effect as observed with conventional tDCS protocols in patients with OCD (Zhou and Fang, 2022) and in the previous case report in hoarding disorder (Handrack et al., 2018). The magnitude of the effects appeared comparable to what we observed at M1 with conventional tDCS on depressive symptoms in patients with treatment-resistant MDD (−25%) (Moirand et al., 2022) and on OC symptoms in patients with OCD (−12%) (Bation et al., 2019).

As cathodal stimulation is thought to have an inhibitory effect on the targeted brain regions, our findings are consistent with evidence-based guidelines which recommend the use of low-frequency repetitive TMS (rTMS), another stimulation technique assumed to have an inhibitory effect, applied over the right DLPFC for patients with OCD (Lefaucheur et al., 2020). In addition, the results on depressive symptoms are consistent with studies indicating the clinical interest of low-frequency rTMS over the right DLPFC in patients with MDD (Brunelin et al., 2014; Lefaucheur et al., 2020).

In the present case study, we report the safety of delivering three 20-min sessions of HD-tDCS per day, spaced 20 min apart, for 5 consecutive days. The development of such protocols is in line with the ongoing trend toward accelerated tDCS protocols for patients with treatment-refractory symptoms (Mondino et al., 2021).

At the neurophysiological level, no significant effects on cortical inhibitory processes were observed using paired-pulse TMS investigation over the motor cortex. However, the clinical changes were accompanied by a marked increase in cortical facilitatory processes. This suggests increased N-methyl-D-aspartate (NMDA) receptor-mediated facilitation after 15 sessions of HD-tDCS. Further physiological studies are needed to elucidate the brain correlates of the clinical effects.

The novelty of the current study lies in the use of a novel tDCS procedure, HD tDCS, instead of the classical tDCS intervention used in the previous case report (Handrack et al., 2018). HD tDCS enables focal and precise stimulation of the targeted cortical region through the use of five small ring electrodes (12 mm external diameter, 4 anodes, 1 cathode). In contrast, conventional tDCS uses two 35 cm^2^ electrodes (1 anode, 1 cathode), stimulating a large cortical region between the two electrodes, as well as deeper structures, and limiting the inference of a causal relationship between the stimulated region and behavior (Kuo et al., 2013; Alam et al., 2016).

Finally, accelerated (3 sessions per day) cathodal HD-tDCS over the right DLPFC seems appropriate and safe for patients with hoarding disorder to reduce hoarding and associated depressive symptoms. Because the significance of the current results is limited by the nature of the study (i.e., uncontrolled, open-label case study in a single patient), further sham-controlled studies are needed to investigate whether such an intervention is meaningful and transferable to routine clinical settings.

## Data availability statement

The original contributions presented in this study are included in this article/Supplementary material, further inquiries can be directed to the corresponding author.

## Ethics statement

Ethical approval was not required for the current study because it involves the off-label use of a medical device in a patient with refractory symptoms and no other therapeutic solution. This treatment was administered at the explicit request of the patient. The study was conducted in accordance with national legislation, the Declaration of Helsinki and institutional requirements. The participant provided written informed consent to participate in this study and received the specified treatment. Written informed consent was obtained from the individual for the publication of any potentially identifiable images or data included in this article.

## Author contributions

JB: Conceptualization, Formal analysis, Investigation, Methodology, Project administration, Supervision, Validation, Writing-original draft. CN: Conceptualization, Formal analysis, Investigation, Methodology, Validation, Visualization, Writing-review and editing. JE: Investigation, Writing-review and editing. FG: Investigation, Writing-review and editing. RB: Conceptualization, Investigation, Writing-review and editing. MM: Conceptualization, Formal analysis, Investigation, Methodology, Project administration, Supervision, Validation, Writing-original draft.
