# Supplementary material for: Case report: accelerated cathodal HD-tDCS over the right dorsolateral prefrontal cortex in hoarding disorder
Source: Front Hum Neurosci. 2024 Mar 11;17:1327811. doi: 10.3389/fnhum.2023.1327811 (PMC10961415; doi:10.3389/fnhum.2023.1327811)
Supplement: Supplementary file 1 [file Table_1.docx]

Supplementary Material

Case report: Accelerated cathodal HD-tDCS over the right dorsolateral prefrontal cortex in hoarding disorder

**Jerome BRUNELIN; Cécilia NEIGE; Julien ECHE; Filipe GALVAO; Rémy BATION; Marine MONDINO**

# Supplementary Table

|  | **rMT** | **spTMS** | **SICI** | | **LICI** | | **ICF** | | **cSP** |
| --- | --- | --- | --- | --- | --- | --- | --- | --- | --- |
|  |  |  | **CS** | **TS** | **CS** | **TS** | **CS** | **TS** |  |
| **%rMT** | 100 | 120 | 80 | 120 | 120 | 120 | 80 | 120 | 130 |
| **%MSO D0** | 51 | 61 | 41 | 61 | 61 | 61 | 41 | 61 | 66 |
| **%MSO D15** | 53 | 64 | 42 | 64 | 64 | 64 | 42 | 64 | 69 |
| **ISI (ms)** |  |  | 2.5 | | 100 | | 10 | |  |

**Table 1: Simulation parameters used to assess corticospinal and intracranial excitability before (D0) and after 15 sessions of HD-tDCS (D15) over the right DLPFC.***rMT: resting motor threshold; spTMS: single-pulse TMS; SICI: short-interval intracortical inhibition; LICI: long-interval intracortical inhibition; ICI: intracortical facilitation; cSP: cortical silent period; MSO: maximum stimulator output; ISI: interval interstimulation.*
